# A case of placental multiple giant chorangioma leading to neonatal death from fetal hydrops

**DOI:** 10.1515/crpm-2022-0008

**Published:** 2022-12-19

**Authors:** Aoi Shiraga, Takuma Ohsuga, Kaoru Kawasaki, Haruta Mogami, Sachiko Minamiguchi, Masaki Mandai

**Affiliations:** Department of Obstetrics and Gynecology, Kyoto University Hospital, Kyoto, Japan; Department of Diagnostic Pathology, Kyoto University Hospital, Kyoto, Japan

**Keywords:** fetal anemia, fetal hydrops, fetal ultrasonography, neonatal death, placental chorangioma

## Abstract

**Objectives:**

Although placental chorangiomas are often asymptomatic, larger tumors (>4–5 cm) can cause various perinatal complications, including polyhydramnios, preterm birth, fetal anemia, fetal hydrops, and intrauterine fetal death. Symptomatic placental chorangiomas are often diagnosed prenatally on ultrasonography as a mass on the fetal side of the placenta.

**Case presentation:**

A 37-year-old pregnant woman underwent emergency cesarean delivery at 34 weeks’ gestation due to rapidly progressive fetal hydrops leading to fetal dysfunction, resulting in neonatal death. Placental pathology indicated multiple placental giant chorangiomas that occupied 40% of the placenta. Because of the disk shape of the placenta, prenatal diagnosis by ultrasonography was difficult.

**Conclusions:**

Some placental chorangiomas are difficult to diagnose and lead to fetal hydrops and poor prognosis, even if ultrasonography does not show an obvious mass in the placenta.

## Introduction

Placental chorangiomas are benign tumors found in about 1% of placentas examined pathologically, with most being small and asymptomatic. Although placental chorangiomas greater than 4–5 cm are very rare (1/500–16,000 pregnancies) [1], about 30% of large chorangiomas are symptomatic and cause various perinatal complications, including polyhydramnios, fetal congestive heart failure, fetal anemia, fetal hydrops, preterm delivery, and sometimes intrauterine fetal death [1, 2]. Large placental chorangiomas, which are associated with poor prognosis, are often diagnosed prenatally using ultrasonography as masses protruding from the fetal side of the placenta [3]. We report a case of multiple giant placental chorangiomas occupying 40% of the placenta that could not be diagnosed prenatally, resulting in neonatal death from fetal hydrops.

## Case presentation

A 37-year-old pregnant woman, gravidity 3, parity 0, with no previous or family history, underwent a prenatal checkup at another hospital, and the pregnancy course was normal. She became aware of decreased fetal movement at 34 weeks 1 day of gestation. On her visit, ultrasonography indicated ascites, pericardial effusion, and cardiomegaly of the fetus. The patient was referred to our hospital at 34 weeks 6 days.

### Examination findings on arrival

**Figure 1:**
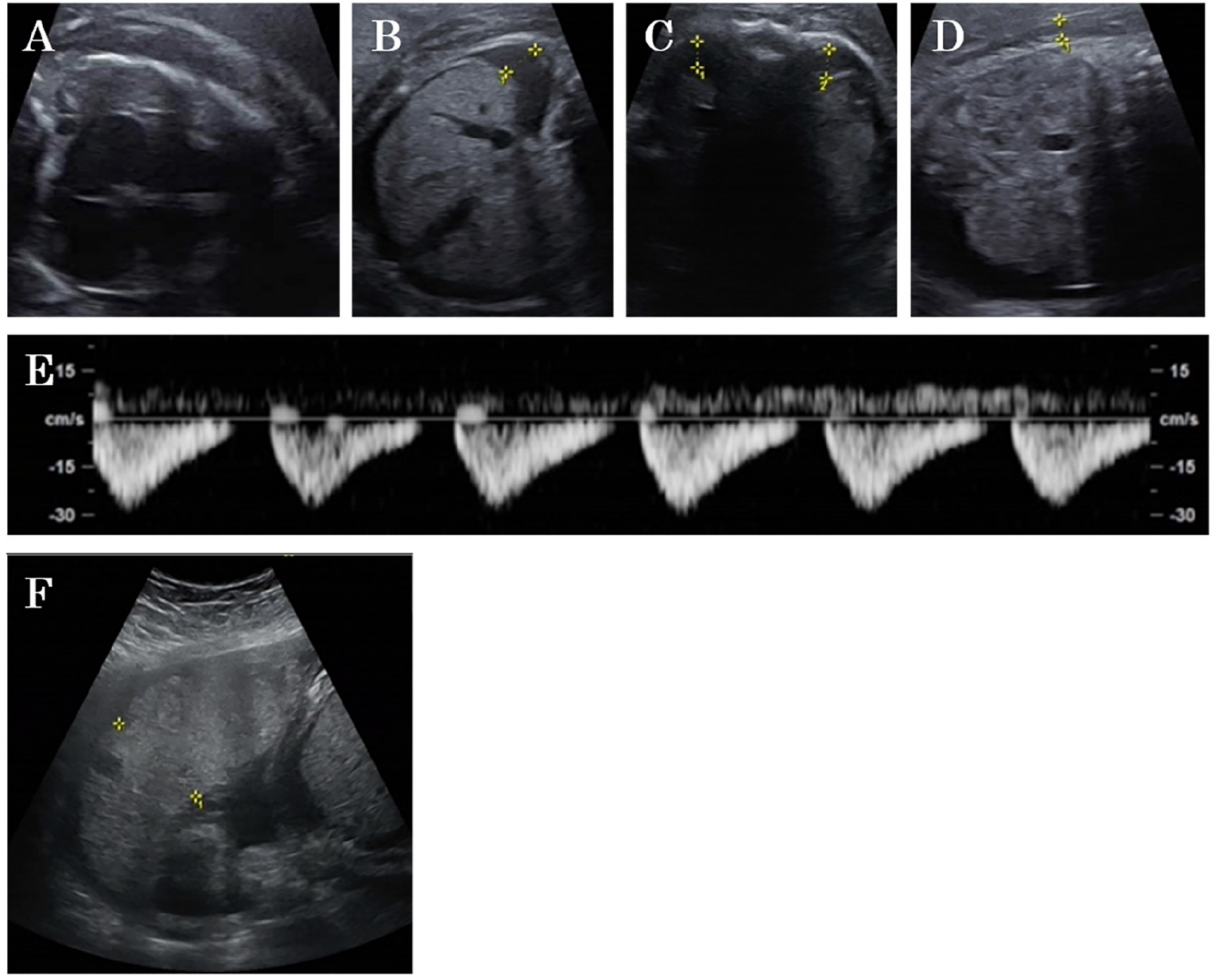
Transabdominal ultrasonography at the first visit. Fetus (tomography): cardiomegaly (A), ascites (B), bilateral pleural effusions (C), and subcutaneous edema (6.4 mm) (D). Umbilical artery (color Doppler methods): absent umbilical artery end-diastolic flow (E) Placenta (tomography): The placenta was not thickened (46 mm), the internal echogenicity was uniform (F), and there was no mass lesion.

The maternal blood test showed type O, Rh+. The other irregular antibodies were negative. Hemoglobin F was 0.3%, and the prothrombin time–international normalized ratio was 0.89. Toxoplasma virus immunoglobulin M (IgM), cytomegalovirus IgM, herpes simplex virus IgM, hepatitis B virus (HBs antigen), hepatitis C virus (HCV antibody), varicella-zoster virus IgM, parvovirus B19 IgM, and syphilis (TP antibody) were all negative. The rubella virus antibody titer was less than eight times higher. Ultrasound examination revealed an estimated fetal weight of 2,257 g (−0.1 SD) without abnormal amniotic fluid volume (amniotic fluid index=18.7 cm), cardiomegaly, tricuspid and mitral regurgitation, bilateral pleural effusions, ascites, and subcutaneous edema were noted (Figure 1A–D). Doppler assessment revealed absent umbilical artery end-diastolic flow (Figure 1E) and pulsation of the umbilical veins. The middle cerebral artery pulsatility index (MCA-PI) and peak systolic velocity (MCA-PSV) were 1.23 and 35.48 cm/s (0.69 MoM), respectively. Regurgitation was noted in the ductus venosus a-wave. Umbilical cord insertion of the placenta was almost central. The thickness of the placenta was 46.4 mm, and it showed heterogeneous brightness and no mass protruding on the fetal side (Figure 1F).

### Cardiotocogram

**Figure 2:**
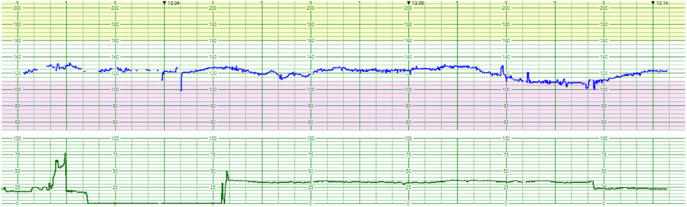
Cardiotocogram at the first visit. Variability was minimal, acceleration was absent, and mild prolonged deceleration was present.

The baseline heart rate was 120 bpm, with minimal variability. There was no acceleration but mild prolonged deceleration, which lasted 4 min at the bottom of 110 bpm. No uterine contractions were detected over a 15 min period (Figure 2).

### Treatment

We made a diagnosis of fetal heart failure, fetal hydrops, and nonreassuring fetal status and the patient underwent emergency cesarean delivery on the same day (34 weeks 6 days). Due to the poor condition of the fetus, priority was given to early delivery, and no further examinations, such as magnetic resonance imaging, were performed.

### Placental pathology

**Figure 3:**
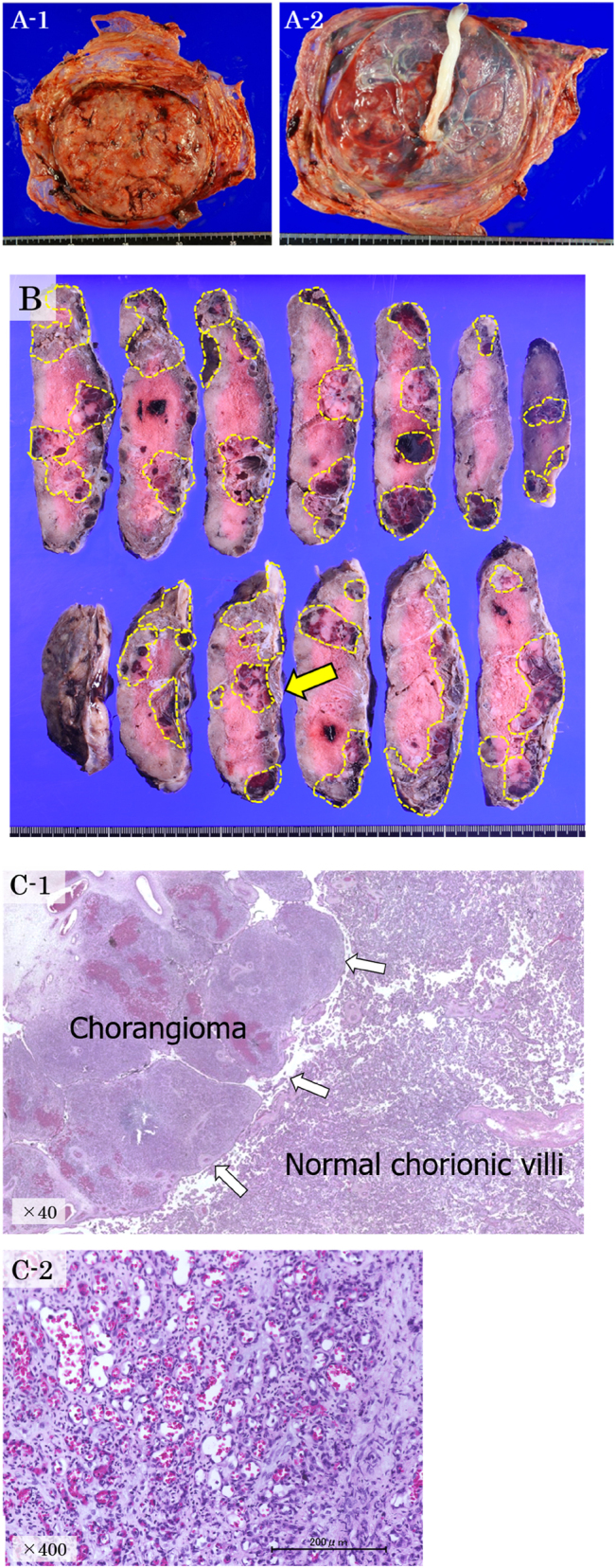
Histopathological examination of the placenta. (A) Gross pathological findings (unfixed): no obvious mass on the maternal (A-1) or fetal surfaces (A-2). Formation of cotyledons is poor. (B) Gross pathological findings (fixed with formalin). There are multiple nodular lesions with hemorrhage or dilated vessels on the cut surfaces, including umbilical cord insertion (with arrow). (C) Histopathological findings: The mass on the left (arrows) shows a well-circumscribed chorangioma. Non-neoplastic chorionic villi can be observed on the right with ischemic changes and collapsed villous stromal vessels (hematoxylin and eosin ×40) (C-1). The nodular lesions are mainly capillary hemangiomatous lesions presenting a proliferation of small vessels with dilatation in some areas. Placental chorangioma was diagnosed (hematoxylin and eosin ×400) (C-2).

Gross pathological findings revealed a large placenta weighing 558 g at the 97th percentile for gestational age with a size of 14 × 13 × 3 cm. The umbilical cord showed two umbilical arteries and one umbilical vein. The cord insertion was in the center, and there was no nodular lesion. There were multiple nodular lesions in the placental disk, with hemorrhage or dilated vessels on the cut surfaces. The largest mass was 10 × 5 × 3 cm, and the mass component occupied approximately 40% of the whole placenta, including the part of the umbilical cord insertion. The color of the nonnodular area was white, showing ischemic change, with poor formation of the cotyledons (Figure 3A, B).

Histopathological findings indicated that the nodular lesions were mainly capillary hemangiomatous lesions presenting a proliferation of small vessels with dilatation in some areas (Figure 3C).

### Progress of the neonate

A 2520 g male was born with Apgar scores of 0 at 1 min, 1 at 5 min (heart rate), and 3 at 10 min (heart rate 2, skin color 1). The pH of the umbilical artery was 6.873, with a base excess of −17.3. The neonate was rigid and had marked subcutaneous edema. No heartbeat was detected after delivery, and we performed resuscitation, including chest compressions. The neonate was admitted to the neonatal intensive care unit. After admission, a blood test showed levels of hemoglobin as 8.9 g/dL, reticulocytes as 41.1‰ and lactate dehydrogenase as 900 U/L, suggesting hemolytic anemia and thrombocytopenia (15,000/L). Intensive care was provided for severe neonatal asphyxia, but he did not respond to high-dose blood transfusions or vasopressors and died at the age of 3 days. A pathological autopsy revealed no external malformations, and the cause of death was diagnosed as cardiac failure and cerebral necrosis associated with placental chorangioma. No other conditions were present, such as blood group incompatibility, infection, or metabolic abnormalities, that would be causative of fetal hydrops.

## Discussion

In the case reported here, placental chorangioma occupied a large part of the placenta, and fetal hydrops rapidly worsened, resulting in hypoxic encephalopathy and neonatal death. Placental chorangiomas larger than 4–5 cm are very rare and cause various perinatal complications [1, 2]. They form an arteriovenous shunt, which increases the circulating blood volume of the fetus, leading to increased fetal urine production and polyhydramnios. In addition, the infant’s cardiac output increases, leading to congestive heart failure. Microvascular damage in the shunt bloodstream results in hemolytic anemia and thrombocytopenia. Heart failure and anemia can cause fetal hydrops, which has a dismal prognosis [1].

Previous studies have reported that poor prognostic factors for placental chorangiomas include tumor size, multiplicity, a tendency for tumor diameter increase, proximity to the umbilical cord insertion, and abundant blood flow within the tumor [1, 3], [4], [5]. This patient had multiple placental chorangiomas with a maximum diameter of 10 cm, including the insertion of the umbilical cord and occupying most of the placenta. There were many poor prognosis factors, and the risk of complications was very high.

Placental chorangiomas large enough to result in poor prognosis are typically diagnosed prenatally. They usually present as nodules in the fetal surface of the placenta on B-mode ultrasound examination and are relatively well-demarcated, hypo-to hyperechoic, internally heterogeneous, substantial masses [3]. In this case, although there were multiple tumors with a maximum diameter of 10 cm, the shape of the placenta remained discoid, and there was no obvious nodule or placental thickening, making diagnosis by ultrasound difficult. Color Doppler examination is characterized by the presence of blood flow inside the mass, which is useful for differentiating it from other placental masses, such as hematomas, degenerative myomas, teratomas, and malignant tumors [1, 3]. In this case, we did not perform color Doppler because of the poor condition of the fetus, and priority was given to delivering the fetus as soon as possible.

This is an exceedingly rare case of multiple placental chorangiomas. To the best of our knowledge, only a few case reports have been published regarding this condition. In a case report by Carlucci et al. in 2021, multiple placental chorangiomas of 3–24 mm were found, with fetal anemia and heart failure appearing at 32 weeks, ultimately resulting in neonatal death after cesarean section [6]. Our review of previous reports of multiple placental chorangiomas showed poor prognosis, with only 4 of 12 cases resulting in live births. In none of the cases was a placental chorangioma suspected based on the shape of the placenta as visible on ultrasound examination. However, in the case reported by Gallot et al. the placenta was carefully monitored by ultrasound, including color Doppler, since the patient had experienced two intrauterine fetal des due to multiple placental chorangiomas in her previous pregnancies [2]. Therefore, although the ultrasound did not demonstrate a typical image of the placental surface protruding into the amniotic cavity, multiple placental chorangiomas were diagnosed prenatally, the baby was delivered at 28 weeks before heart failure, and the infant showed good progress after birth. Although it is difficult to diagnose multiple placental chorangiomas based on the shape of the placenta, early diagnosis and strict follow-up of the fetus’s condition may improve prognosis.

When placental chorangiomas are diagnosed prenatally, frequent follow-up is important for evaluating the tumor and the condition of the fetus. The evaluation of fetal blood flow using ultrasound color Doppler and pulsed Doppler methods is useful for assessing the fetus’s condition [5, 7]. In this case, fetal blood flow was abnormal, with absent umbilical artery end-diastolic flow and reversed a-wave in the ductus venosus, reflecting a poor prognosis. Although the neonate had anemia, the prenatal MCA-PSV was not elevated at 35.48 cm/s (0.69 MoM). We presume that this case had rapidly progressive cardiac failure and anemia resulting from tumor growth, and the MCA-PSV might not have been high because of the failure of the compensatory function of cerebral blood circulation caused by severe fetal circulatory failure.

Severe complications often occur in the late second trimester [8]. Treatments, such as amniodrainage, are used to reduce the volume of amniotic fluid, and fetal transfusion may be an option for continuing the pregnancy due to fetal immaturity. The embolization of the shunt vessel or endoscopic laser coagulation might also be useful [5]. There exists no definite standard for delivery in cases where fetal hydrops develop due to placental chorangioma in the second trimester, with the choices including conservative treatment, fetal treatment, or delivery, depending on the number of gestation weeks and fetal hydrops severity. According to a systematic review reported in 2020, a 40.5% rate of perinatal mortality was observed in cases with fetal hydrops due to placental chorangioma. Of the total, 42.5% underwent fetal treatment, such as laser coagulation or alcohol embolization, of which 57.3% demonstrated improvement in fetal hydrops or abnormal blood flow and 28.9% showed no improvement. Among the patients whose fetus received treatment, perinatal mortality and intrauterine fetal mortality were 31.2 and 23.6%, respectively [9]. Therefore, even when fetal hydrops develops, the neonate’s prognosis might improve depending on the delivery timing. There is a lack of evidence regarding which cases should be treated with fetal therapy or delivered, and more cases need to be accumulated and examined. When fetal hydrops occurs after 34 weeks, delivery is generally recommended and is not limited to those caused by placental chorangioma [10].

The possibility of fetomaternal transfusion syndrome should also be considered when fetal anemia is present. In this case, fetomaternal transfusion syndrome was negative because the maternal blood test did not show hemoglobin F (0.3%) elevation and the neonatal blood test suggested hemolytic anemia.

## Conclusions

In this case, placental chorangiomas occupied a large portion of the placenta, and we presume that fetal hydrops progressed rapidly, leading to hypoxic encephalopathy and neonatal death. Despite the presence of multiple giant placental chorangiomas, the placenta was disk-shaped with no protruding masses on the surface. Even among cases with an absence of an obvious placental mass on ultrasonography, some placental chorangiomas result in fetal hydrops and poor prognosis. If placental chorangioma is suspected at an early stage, it is important to evaluate the placenta for poor prognostic factors, the condition of the fetus, and the timing of delivery, while taking into consideration the possibility of rapid changes in the fetal circulatory dynamics. Furthermore, in the evaluation of fetal edema, it is important to evaluate the placenta by color Doppler examination, even among cases with an absence of an obvious placental mass on B mode ultrasound examination.

## Supplementary Material

Supplementary Material
