# Supplementary material for: A case of placental multiple giant chorangioma leading to neonatal death from fetal hydrops
Source: Case Rep Perinat Med. 2022 Dec 19;12(1):20220008. doi: 10.1515/crpm-2022-0008 (PMC11616974; doi:10.1515/crpm-2022-0008)
Supplement: Supplementary file 1 — Supplementary Material [file j_crpm-2022-0008_suppl.pdf]

Manuscript no. ....

LICENSE TO PUBLISH

Please read the terms of this agreement, print, sign, scan and submit the document to the online submission system of this journal. Case Reports in Perinatal Medicine ("Journal"), De Gruyter GmbH ("Journal Owner")

Article entitled ("Work" or "article"): A case of placental multiple giant chorangioma leading to neonatal death from fetal hydrops

Author(s) (also referred to as "Licensor(s)"): Aoi Shiraga, Takuma Ohsuga, Kaoru Kawasaki, Haruta Mogami, Sachiko Minamiguchi, Masaki Mandai

Corresponding author: (if more than one author): Takuma Ohsuga.

1. **License**

The use of the article will be governed by the Creative Commons Attribution license as currently displayed on http://creativecommons.org/licenses/by/4.0, except that sections 2 through 7 below will apply in this respect and prevail over all conflicting provisions of such license mode, effective upon acceptance for publication.

2. **Author's Warranties**

The author warrants that the article is original, written by stated author/s, has not been published before, contains no unlawful statements, does not infringe the rights of others, is subject to copyright that is vested exclusively in the author and free of any third party rights, and that any necessary written permissions to quote from other sources have been obtained by the author(s).

3. **User Rights**

Under the Creative Commons Attribution license, the users are free to share (copy, distribute and transmit the contribution) and adapt (remix, transform, and build upon the material) the article for any purpose, even commercially, provided they attribute the contribution in the manner specified by the author or licensor.

4. **Rights of Authors**

Authors retain the following rights:

- copyright, and other proprietary rights relating to the article, such as patent rights,
- the right to use the substance of the article in future own works, including lectures and books,
- the right to reproduce the article for own purposes,
- the right to self-archive the article.

5. **Co-Authorship**

If the article was prepared jointly with other authors, the signatory of this form warrants that he/she has been authorized by all co-authors to sign this agreement on their behalf, and agrees to inform his/her co-authors of the terms of this agreement.

6. **Royalties**

This agreement entitles the author to no royalties or other fees. To such extent as legally permissible, the author waives his or her right to collect royalties relative to the article in respect of any use of the article by the Journal Owner or its sublicensee.

7. **Miscellaneous**

The Journal Owner will publish the article (or have it published) in the Journal, if the article's editorial process is successfully completed and the Journal Owner or its sublicensee has become obligated to have the article published. Where such obligation depends on the payment of a fee, it shall not be deemed to exist until such time as that fee is paid. The Journal Owner may conform the article to a style of punctuation, spelling, capitalization and usage that it deems appropriate. The author acknowledges that the article may be published so that it will be publicly accessible and such access will be free of charge for the readers. The Journal Owner will be allowed to sublicense the rights that are licensed to it under this agreement.

The Journal Owner will be entitled to enforce in respect of third parties, to such extent as permitted by law, the rights licensed to it under this agreement.

If the article was written in the course of employment by the US or UK Government, and/or arises from NIH funding, please consult the Journal Owner for further explanation.

Author's Signature: Aoi Shiraga

Name printed: Aoi Shiraga

Date: March 4th, 2022
